# Upregulation of *Reg IV* and *Hgf* mRNAs by Intermittent Hypoxia via Downregulation of microRNA-499 in Cardiomyocytes

**DOI:** 10.3390/ijms232012414

**Published:** 2022-10-17

**Authors:** Shin Takasawa, Asako Itaya-Hironaka, Mai Makino, Akiyo Yamauchi, Sumiyo Sakuramoto-Tsuchida, Tomoko Uchiyama, Ryogo Shobatake, Yoshinori Takeda, Hiroyo Ota

**Affiliations:** 1 Department of Biochemistry, Nara Medical University, 840 Shijo-cho, Kashihara 634-8521, Nara, Japan; 2 Department of Diagnostic Pathology, Nara Medical University, 840 Shijo-cho, Kashihara 634-8522, Nara, Japan; 3 Department of Respiratory Medicine, Nara Medical University, 840 Shijo-cho, Kashihara 634-8522, Nara, Japan

**Keywords:** intermittent hypoxia, sleep apnea syndrome, sustained hypoxia, Reg IV, HGF, microRNA-499

## Abstract

Sleep apnea syndrome (SAS) is characterized by recurrent episodes of oxygen desaturation and reoxygenation (intermittent hypoxia [IH]), and is a risk factor for cardiovascular disease (CVD) and insulin resistance/Type 2 diabetes. However, the mechanisms linking IH stress and CVD remain elusive. We exposed rat H9c2 and mouse P19.CL6 cardiomyocytes to experimental IH or normoxia for 24 h to analyze the mRNA expression of several cardiomyokines. We found that the mRNA levels of regenerating gene IV (*Reg IV*) and hepatocyte growth factor (*Hgf*) in H9c2 and P19.CL6 cardiomyocytes were significantly increased by IH, whereas the promoter activities of the genes were not increased. A target mRNA search of microRNA (miR)s revealed that rat and mouse mRNAs have a potential target sequence for miR-499. The miR-499 level of IH-treated cells was significantly decreased compared to normoxia-treated cells. MiR-499 mimic and non-specific control RNA (miR-499 mimic NC) were introduced into P19.CL6 cells, and the IH-induced upregulation of the genes was abolished by introduction of the miR-499 mimic, but not by the miR-499 mimic NC. These results indicate that IH stress downregulates the miR-499 in cardiomyocytes, resulting in increased levels of *Reg IV* and *Hgf* mRNAs, leading to the protection of cardiomyocytes in SAS patients.

## 1. Introduction

Sleep apnea syndrome (SAS) is a common disorder characterized by repetitive episodes of oxygen desaturation during sleep, the development of daytime sleepiness, and the deterioration of the patient’s quality of life [1,2]. SAS leads to intermittent hypoxia (IH) [3,4], hypercapnia, and subsequent reoxygenation, as well as disruption of sleep architecture such as sleep fragmentation. SAS has been reported to affect middle-aged and older individuals, with the prevalence estimated to be around 22% in men and 17% in women [5]. SAS is associated with many systemic complications, such as obesity; type 2 diabetes [6,7]; dyslipidemia [8]; cardiovascular diseases, including hypertension, coronary disease, heart failure, and stroke [9,10,11]; pulmonary hypertension [12]; neurocognitive deficits [13,14]; depression [15]; and impaired memory [16].

Observational studies have indicated that SAS is associated with a high risk of serious cardiovascular disease (CVD), including sudden death, atrial fibrillation, stroke, and coronary artery disease, leading to heart failure. It has been reported that SAS is a major independent risk factor for CVD, such as systemic and pulmonary hypertension, congestive heart failure, and stroke [17], as well as myocardial infarction, cerebrovascular dysfunction, and idiopathic sudden death [9]. IH-induced cardiomyocyte damage occurs with the increases of intracellular reactive oxygen species during reoxygenation following hypoxia [18,19,20]. Moreover, IH may cause lipid peroxidation [21], protein oxidation, DNA damage [22], and attenuation of antioxidant enzyme capacity, thus reducing cardiomyocyte numbers by cell death [23]. The prevalence of SAS in patients with heart failure ranges from 15% to 59%, and the mortality rate of patients with severe SAS is significantly high [24,25,26,27]. In addition, cardiac function is impaired with left ventricular hypertrophy in obese patients with severe SAS [28]. Hypertension, cardiac remodeling, and other complications of SAS have been studied using rodent models of IH [29].

In this study, we investigated the direct effect of IH, a hallmark of SAS, using rat and mouse cardiomyocytes and an in vitro IH system. For in vitro IH, nitrogen and oxygen are delivered by a controlled system that regulates the flow of gases. We investigated the direct effect of IH on the gene expression of cytokines and cardiac protective/regenerative factors, such as regenerating gene (*Reg*) family genes and hepatocyte growth factor (*Hgf*). Significant increases in the mRNA levels of *Reg IV* and *Hgf*, which both generate growth factors with proliferative and anti-apoptotic effects, were detected in rat and mouse cardiomyocytes in response to IH treatment via the downregulation of microRNA (miR)-499.

## 2. Results

### 2.1. Gene Expressions of Reg IV and Hgf Were Increased by IH in Cardiomyocytes

**Figure 1:**
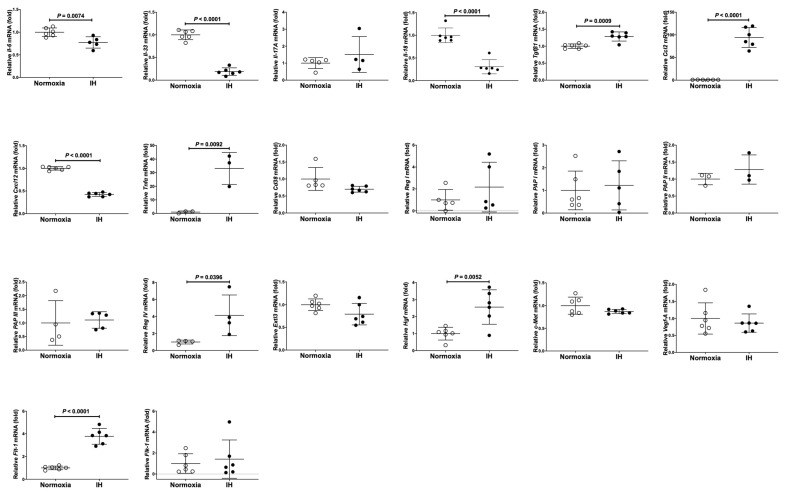
The mRNA levels of rat *Il-6*, *Il-33*, *Il-17A*, *Il-18*, *Tgfβ1*, *Cxcl12*, *Tnfα*, *Ccl2*, *Vegf-A*, *Flt-1*, *Flk-1*, *Cd38*, *Reg I*, *PAP I*, *PAP II*, *PAP III*, *Reg IV*, *Extl3*, *Hgf*, and *c-Met* in rat H9c2 cardiomyocytes. Rat H9c2 cells were treated with normoxia or IH for 24 h. The mRNA levels were measured by real-time RT-PCR and normalized by *rat insulinoma gene* (*Rig*)*/ribosomal protein S15* (*RpS15*) as an internal standard. The mRNA levels exposed to normoxia were set to 1.0. Open and closed circles indicate values of relative mRNA expression of cells exposed to normoxia and IH, respectively. Data are expressed as the mean ± SD of the samples. Statistical analyses were performed using Student’s *t*-test. IH significantly increased the mRNA levels of *Tgfβ1*, *Tnfα*, *Ccl2*, *Flt-1*, *Hgf*, and *Reg IV* in rat H9c2 cells. IH significantly decreased the mRNA levels of *Il-6*, *Il-33*, *Il-18*, and *Cxcl12* in rat H9v2 cells. The other gene expressions (*Il-17A*, *Cd38*, *Reg I*, *PAP I*, *PAP II*, *PAP III*, *Extl3*, *c-Met*, *Vegf-A*, and *Flk-1*) did not show significant changes.

**Figure 2:**
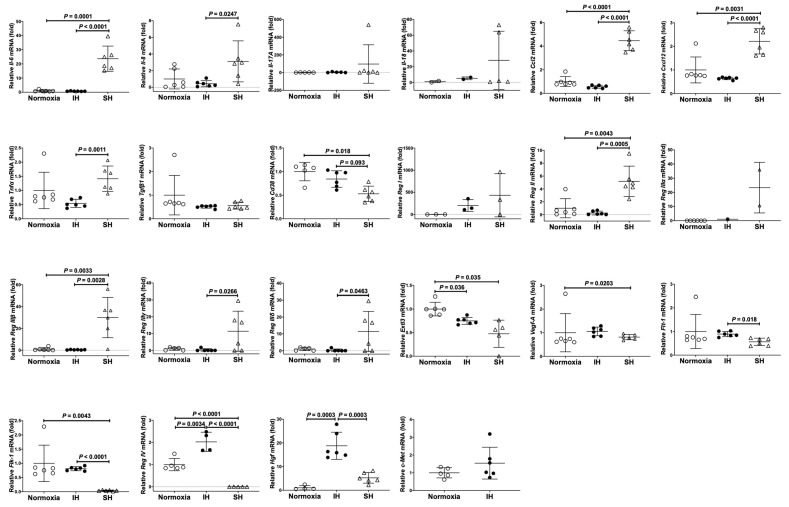
The mRNA levels of mouse *Il-6*, *Il-8*, *Il-17A*, *Il-18*, *Tgfβ1*, *Ccl2*, *Cxcl12*, *Tnfα*, *Vegf-A*, *Flt-1*, *Flk-1*, *Cd38*, *Reg I*, *Reg II*, *Reg IIIα*, *Reg IIIβ*, *Reg IIIγ*, *Reg IIIδ*, *Reg IV*, *Extl3*, *Hgf*, and *c-Met* in mouse P19.CL6 cardiomyocytes. Mouse P19.CL6 cells were treated with normoxia, IH, or SH for 24 h. The mRNA levels were measured by real-time RT-PCR and normalized by *Rig/RpS15* as an internal standard. The mRNA levels exposed to normoxia were set to 1.0. Open and closed circled circles indicate values of relative mRNA expression of cells exposed to normoxia and IH, respectively. Data are expressed as the mean ± SD of the samples. Statistical analyses were performed using Student’s *t*-test. IH significantly increased the mRNA levels of *Reg IV* and *Hgf* in mouse P19.CL6 cells. SH significantly increased the mRNA levels of *Il-6*, *Il-8*, *Ccl2*, *Cxcl12*, *Tnfα*, *Reg II*, *Reg IIIβ*, *Reg IIIγ*, and *Reg IIIδ* in P19.CL6 cells. IH and/or SH decreased mRNA levels of *Cd38*, *Extl3*, *Vegf-A*, *Flt-1*, and *Flk-1*. The other gene expressions (*Il-17A*, *Il-18*, *Tgfβ1*, *Reg I*, *Reg IIIα*, and *c-Met*) did not show significant changes.

We exposed rat H9c2 cardiomyocytes and cardiomyocytic differentiated mouse P19.CL6 cells to normoxia, IH, or sustained hypoxia (SH) for 24 h. After the treatment, we measured the mRNA levels of cardiomyocytic inflammation related interleukin genes, chemokine genes, cytokine genes), genes of cardiomyocytic growth/regeneration factors and receptors, and genes of cardiomyocyte functioning: *interleukin (Il)-6*, *Il-17A*, *Il-18*, *Il-33*, *transforming growth factor (Tgf)β1*, *C-C motif chemokine ligand 2* (*Ccl2*), *C-X-C motif chemokine ligand 12* (*Cxcl12*), *tumor necrosis factor-α* (*Tnfα*), *vascular endothelial growth factor A* (*Vegf-A*), *Fms-like tyrosine kinase 1* (*Flt-1*: *Vegf receptor* [*Vegfr*] *1*), *fetal liver kinase receptor 1* (*Flk-1*), *cluster of differentiation 38* (*Cd38*: encoding ADP-ribosyl cyclase/cyclic ADP-ribose hydrolase), *Reg I*, *pancreatitis associated protein* (*PAP*) *I*, *PAP II*, *PAP III*, *Reg IV*, *Exostosin-like 3* (*Extl 3*)/*Reg receptor*, *Hgf*, and *tyrosine-protein kinase Met* (*c-Met*: encoding Hgf receptor) in rat H9c2 cells. We measured mRNA levels of *Il-6*, *Il-8*, *Il-17A*, *Il-18*, *Tgf-β1*, *Ccl2*, *Cxcl12*, *Tnfα*, *Vegf-A*, *Flt-1*, *Flk-1*, *Cd38*, *Reg I*, *Reg II*, *Reg IIIα*, *Reg IIIβ*, *Reg IIIγ*, *Reg IIIδ*, *Reg IV*, *Extl3*, *Hgf*, and *c-Met* in mouse P19.CL6 cells by using real-time reverse transcriptase-polymerase chain reaction (RT-PCR). As shown in Figure 1, significant increases in *Tgfβ1*, *Ccl2*, *Tnfα*, *Flt-1*, *Reg IV*, and *Hgf* were detected in IH-treated rat H9c2 cells. However, *Tgf-β1*, *Ccl2*, *Tnfα*, and *Flt-1* were not specifically increased by IH in mouse P19.CL6 cardiomyocytes. In contrast, the mRNAs of *Reg IV* and *Hgf* were significantly and specifically increased by IH in mouse P19.CL6 cells (Figure 2). 

**Figure 3:**
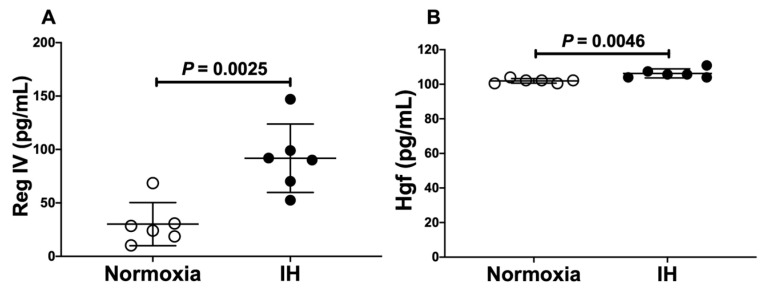
Concentrations of (**A**) Reg IV and (**B**) Hgf in a mouse P19.CL6 cardiomyocyte culture medium. P19.CL6 cardiomyocytes were treated by normoxia or IH condition for 24 h. The concentrations of Reg IV and Hgf were measured by ELISA. Open and closed circles indicate values of culture medium of cells exposed to normoxia and IH, respectively. Data are expressed as mean ± SD for each group. The statistical analyses were performed using Student’s *t*-test.

We further measured Reg IV and Hgf proteins in the culture medium of differentiated P19.CL6 cells by the enzyme-linked immunosorbent assay (ELISA). We found that the levels of Reg IV and Hgf were significantly increased by IH (Reg IV [30.16 pg/mL vs. 91.83 pg/mL, *p* = 0.0025], and Hgf [101.9 pg/mL vs. 106.3 pg/mL, *p* = 0.0046]) (Figure 3).

### 2.2. Reg IV and Hgf Act as Autocrine/Paracrine Growth and Anti-Apoptotic Factors in SH/IH Condition(s) for Cardiomyocytes

**Figure 4:**
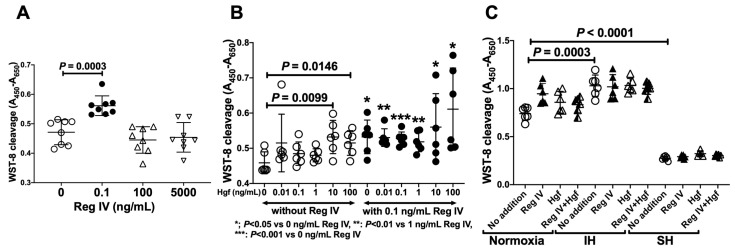
Cardiomyocyte proliferation by Reg IV and Hgf. (**A**) Mouse recombinant Reg IV (0–5000 ng/mL) was added to a differentiated mouse P19.CL6 cardiomyocyte culture medium in SH condition, and cell numbers were measured by WST-8 assay. Open circles, closed circles, upward open triangles, and downward pointing open triangles indicate WST-8 values of the cells in the addition of 0, 0.1, 100, and 5000 ng/mL Reg IV, respectively. (**B**) Hgf (0–100 ng/mL) was added in a differentiated mouse P19.CL6 cell culture medium, and the cells were incubated for 24 h in SH condition. Viable cell numbers were measured by a WST-8 assay. (**C**) The cells were incubated in normoxia, IH, or SH in the presence/absence of Reg IV (0.1 ng/mL) and/or Hgf (0.1 ng/mL) for 24 h. Viable cell numbers were measured by a WST-8 assay. Data are expressed as mean ± SD for each group. The statistical analyses were performed using Student’s *t*-test.

To evaluate the direct effects of Reg IV and Hgf on cardiomyocyte proliferation, differentiated P19.CL6 cells were incubated with Reg IV and Hgf for 24 h. Following the SH treatment, cell viability was determined by using a WST-8 (2-[2-methoxy-4-nitrophenyl]-3-[4-nitrophenyl]-5-[2,4-disulfophenyl]-*2H*-tetrazolium monosodium salt) assay. P19.CL6 cell proliferation was significantly increased by 0.1 ng/mL Reg IV (Figure 4A) and 10–100 ng/mL Hgf (Figure 4B), and the 0.1 ng/mL Reg IV-induced proliferation was further enhanced by the combined addition of Hgf (Figure 4B). P19.CL6 cell numbers were significantly increased by IH and dramatically reduced by SH (Figure 4C).

**Figure 5:**
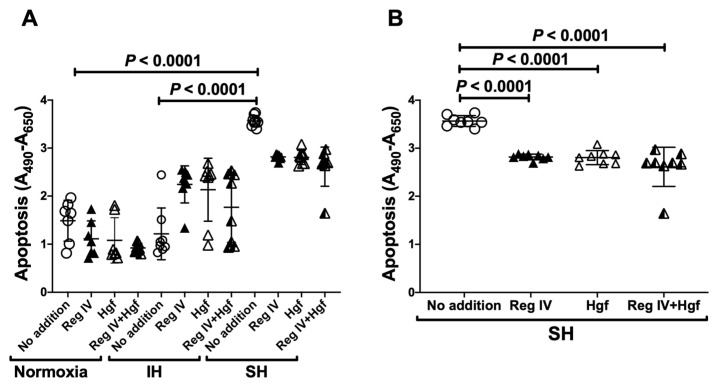
(**A**) Anti-apoptotic effects of Reg IV and Hgf in differentiated P19.CL6 cardiomyocytes in normoxia, IH, or SH. IH did not increase apoptosis and SH increased apoptosis (*p* < 0.0001 vs. normoxia; *p* < 0.0001 vs. IH). (**B**) Anti-apoptotic effects of Reg IV and/or Hgf in SH. Apoptosis was quantified using the TUNEL method. Mouse recombinant Reg IV (0.1 ng/mL) and Hgf (0.1 ng/mL) were added to differentiated mouse P19.CL6 cell culture medium and incubated in normoxia, IH, or SH for 24 h. Data are expressed as mean ± SD for each group. The statistical analyses were performed using Student’s *t*-test.

To see why the cell numbers were reduced by SH, we also measured apoptosis of IH/SH-stimulated P19.CL6 cells using the TUNEL (TdT-mediated dUTP nick end labeling) method. We found that SH stimulation significantly increased cell apoptosis compared to normoxia/IH (Figure 5A) and that the addition of Reg IV and Hgf in the cultured medium in SH significantly reduced the apoptosis (Figure 5B).

**Figure 6:**
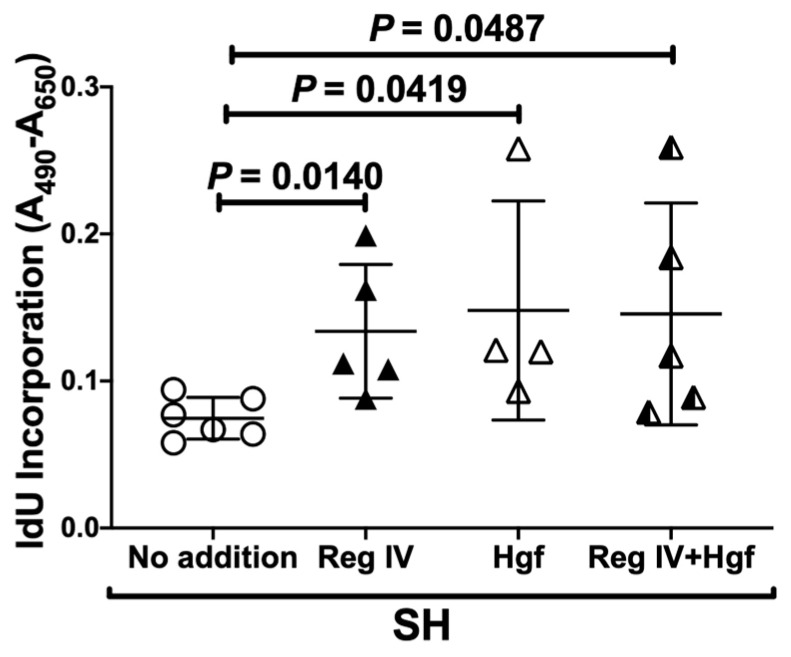
Replicative DNA synthesis of mouse P19.CL6 cardiomyocytes incubated in SH in the presence/absence of Reg IV (0.1 ng/mL) and/or Hgf (0.1 ng/mL). P19.CL6 cells were exposed to SH (1% O_2_) for 24 h, and replicated DNA synthesis was measured by IdU incorporation. Data were expressed as mean ± SD for each group. The statistical analyses were performed using Student’s *t*-test.

We then measured the replicative DNA synthesis of SH-treated P19.CL6 cells by 5-iodo-2′-deoxyuridine (IdU: pyrimidine analog) incorporation. As shown in Figure 6, replicative DNA synthesis was significantly increased by the addition of Reg IV and/or Hgf in SH-treated cardiomyocytes.

The results fitted well with those of previous papers which reported that Reg protein and Hgf functioned as anti-apoptotic and growth/differentiation factors for cardiomyocytes [30,31,32,33,34,35], and that Hgf acts as an anti-apoptotic factor against high concentration Reg-induced apoptosis [36].

### 2.3. The Promoter Activities of Reg IV and Hgf Were Not Increased by IH

**Figure 7:**
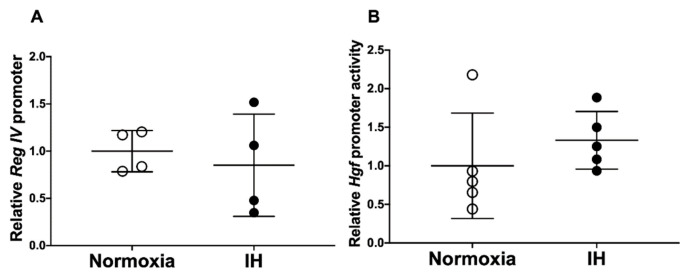
Luciferase assays of promoter activities of (**A**) *Reg IV* and (**B**) *Hgf* in P19.CL6 cells. Reporter plasmids, prepared by inserting the promoter fragments of mouse *Reg IV* (−2008–+29) upstream of a firefly luciferase reporter gene in pGL4.17 vector and rat *Hgf* (−1336–+59) in pGL3-Basic vector [36], were transfected into P19.CL6 cells. After the cells were exposed to either IH or normoxia for 24 h, the cells were lysed, and the promoter activities of *Reg IV* and *Hgf* were measured. The promoter activity was normalized for variations in transfection efficiency using β-galactosidase activity as an internal standard. The promoter activities exposed to normoxia were set to 1.0. All data are represented as the mean ± SD of the samples. The statistical analyses were performed using Student’s *t*-test.

To determine whether the IH-induced increases in *Reg IV* and *Hgf* mRNAs were caused by the activation of transcription, a 2037 bp fragment containing 2008 bp of the mouse *Reg IV* promoter was fused to the luciferase gene of pGL4.17. The mouse *Reg IV* promoter construct and the rat *Hgf* promoter construct, which had a 1395 bp fragment containing 1336 bp of the rat *Hgf* promoter inserted into a pGL3-Basic vector [36], were transfected into differentiated P19.CL6 cells. After IH stimulation, the promoter activities of *Reg IV* and *Hgf* were measured. We found that *Reg IV* and *Hgf* promoter activities were not activated by IH in the differentiated P19.CL6 cells (Figure 7: *p* = 0.6289 and *p* = 0.3407, respectively). These results suggested that the gene expression of *Reg IV* and *Hgf* in response to IH was not regulated by transcription.

### 2.4. The miR-499 Level Was Significantly Decreased by IH

**Figure 8:**
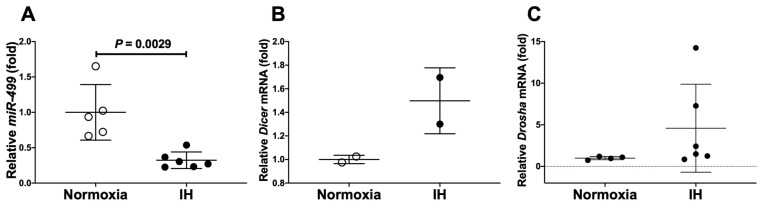
The levels of (**A**) *miR-499*, (**B**) *Dicer* mRNA, and (**C**) *Drosha* mRNA of P19.CL6 cells treated with normoxia or IH for 24 h. The levels of *miR-499* and *Dicer* and *Drosha* mRNAs were measured by real-time RT-PCR using *U6* (for *miR-499*) and *Rig/RpS15* (for *Dicer* and *Drosha*) as endogenous controls. The miR-499/mRNA levels exposed to normoxia were set to 1.0. Data are expressed as mean ± SD for each group. The statistical analyses were performed using Student’s *t*-test.

We considered the possible explanation that IH-induced up-regulation of *Reg IV* and *Hgf* was controlled post-transcriptionally. Therefore, we searched the targeted miRNA using the MicroRNA.org program (http://www.microrna.org/microrna/home.do, accessed on 29 October 2021), which revealed that *Reg IV* and *Hgf* mRNAs have a potential target sequence for miR-499. There were no other miRNA candidates targeting both genes. We measured the miR-499 levels of IH-treated cells by RT-PCR and found that the level was significantly lower than that of normoxia-treated cells (0.3229 folds vs. normoxia, *p* = 0.0029). The possible reasons as to why the level of miR-499 was decreased by IH include the following: mRNA levels of some enzymes involved in miRNA biosynthesis are influenced by IH, and the level of miR-499 was specifically decreased by IH either via decreased biosynthesis or enhanced degradation. We measured the mRNA levels of *ribonuclease type III* (*Drosha*) and *endoribonuclease Dicer* (*Dicer*), which are involved in the biosynthesis of miRNAs [37,38] and found that their expression was unchanged by IH (Figure 8: *p* = 0.2200 and *p* = 0.1299, respectively).

**Figure 9:**
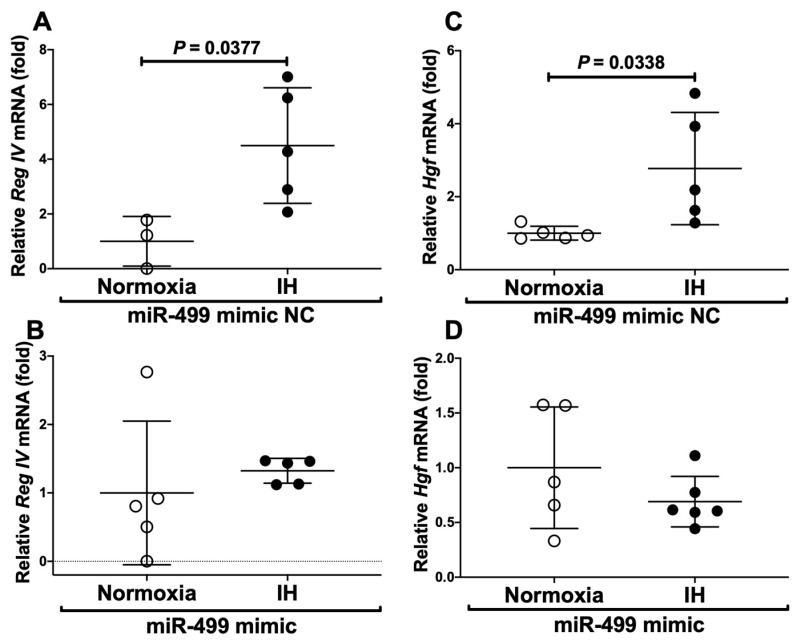
Effects of miR-499 mimic transfection on *Reg IV* and *Hgf* expression. The miR-499 mimic (5′-UUAAGACUUGCAGUGAUGUUU-3′, 5′-ACAUCACUGCAAGUCUUAAuu-3′) and non-specific control RNA (miR-499 mimic NC) (5′-UUCUCCGAACGUGUCACGUtt-3′, 5′-ACGUGACACGUUCGGAGAAtt-3′) were synthesized by the Nihon Gene Research Laboratories, Inc. (NGRL; Sendai, Japan) and introduced into differentiated P19.CL6 cells using Lipofectamine^®^ RNAiMAX just before IH/normoxia exposure. The mRNA levels of *Reg IV* and *Hgf* were measured by real-time RT-PCR, as described in the Materials and Methods section. The expression of *Reg IV* and *Hgf* mRNA were measured by real-time RT-PCR, using *Rig/RpS15* as an endogenous control. The mRNA levels exposed to normoxia were set to 1.0. The figure represents (**A**) *Reg IV* mRNA expression in miR-499 mimic NC-introduced cells, (**B**) *Reg IV* mRNA expression in miR-499 mimic-introduced cells, (**C**) *Hgf* mRNA expression in miR-499 mimic NC-introduced cells, and (**D**) *Hgf* mRNA expression in miR-499 mimic-introduced cells. Data are expressed as mean ± SD for each group. The statistical analyses were performed using Student’s *t*-test.

**Figure 10:**
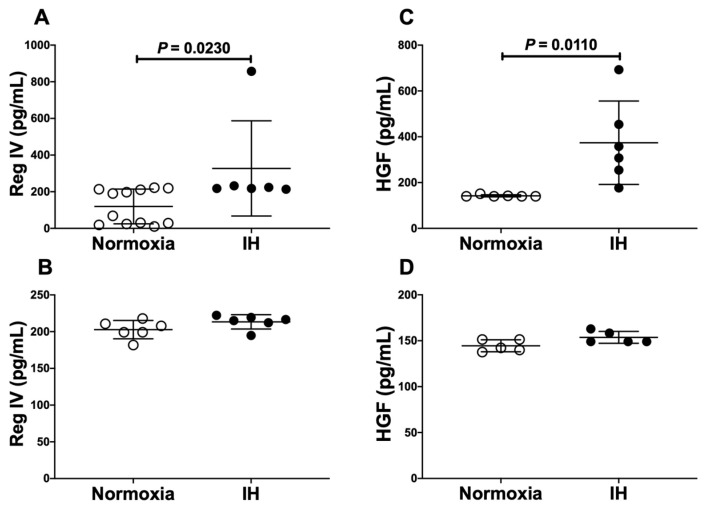
The effects of miR-499 mimic transfection on Reg IV and Hgf expression. Concentrations of medium Reg IV and Hgf were measured by ELISA as described in the Materials and Methods section. The figure represents (**A**) Reg IV expression in miR-499 mimic NC-introduced cells, (**B**) Reg IV expression in miR-499 mimic-introduced cells, (**C**) Hgf expression in miR-499 mimic NC-introduced cells, and (**D**) Hgf expression in miR-499 mimic-introduced cells. Data are expressed as mean ± SD for each group. The statistical analyses were performed using Student’s *t*-test.

These results suggest that miR-499 plays a key role in the post-transcriptional regulation of mRNA levels of *Reg IV* and *Hgf*. To investigate whether *Reg IV* and *Hgf* expression in IH is regulated by miR-499, miR-499 mimic and non-specific control RNA (miR-499 mimic NC) were introduced into differentiated P19.CL6 cells with IH/normoxia exposure, and the mRNA levels of *Reg IV* and *Hgf* were measured by real-time RT-PCR. As shown in Figure 9 and Figure 10, we found that IH-induced increases in *Reg IV* and *Hgf* mRNAs, and IH-induced increases in Reg IV and Hgf in the culture medium, were abolished by the introduction of the miR-499 mimic, but not by the miR-499 mimic NC. These findings indicate that IH stress downregulated the miR-499 level in cardiomyocytes (Figure 8), and that the levels of *Reg IV* and *Hgf* mRNAs were increased via a miR-499 mediated mechanism.

## 3. Discussion

In this study, we demonstrated that IH exposure induced increases in *Reg IV* and *Hgf* mRNA levels, and that Reg IV and Hgf functioned as anti-apoptotic factor(s) in hypoxia (SH/IH)-exposed cardiomyocytes. We further studied the mechanisms by which IH upregulates the mRNA levels of *Reg IV* and *Hgf* and found the possibility of post-transcriptional miRNA-regulated mechanisms in which miR-499 is involved.

*Reg* was first found in regenerating pancreatic islets [39] and its β-cell replication activity in vitro and in vivo was clarified [39,40,41]. The *Reg* and *Reg*-related genes were isolated and revealed to comprise a multigene family, the *Reg* gene family [42,43]. Based on the primary structures of the Reg proteins, the members of the family are grouped into four subclasses: types I, II, III, and IV [43]. In humans, four *REG* family genes (i.e., *REG Iα* [39,44], *REG Iβ* [45], *REG*-related sequence (*RS*: pseudogene) [44], *HIP* [46]/*PAP* [47], and *REG III* [48]) are tandemly ordered in the 95 kbp region of chromosome 2p12 [49], whereas *REG IV* is located on chromosome 1 [50]. In the mouse genome, all the *Reg* family genes, except for *Reg IV*, (i.e., *Reg I*, *Reg II*, *Reg IIIα*, *Reg IIIβ*, *Reg IIIγ*, and *Reg IIIδ*) have been mapped to a contiguous 75 kbp region of chromosome 6C [51], whereas *Reg IV* has been mapped on chromosome 3. Type I (and Type II) Reg proteins are expressed in regenerating islets [39,52] and involved in β-cell regeneration [3,40,53,54,55,56,57,58,59,60,61]. It has been suggested that Reg family proteins are involved in cellular proliferation in exocrine pancreatic cells [62,63], gastrointestinal cells [64,65,66,67,68,69,70,71,72,73,74,75], hepatic cells [76,77,78,79,80], salivary ductal cells [81,82,83], bone and muscle cells [84,85], neuronal cells [86], and cardiovascular cells [30,32,87,88].

HGF is well known as a mesenchyme-derived multifunctional protein that plays a critical role in cell survival, proliferation, migration, and differentiation [89]. Earlier studies demonstrated that the HGF receptor, a receptor tyrosine kinase, encoded by the c-*met* proto-oncogene, was expressed in various cells of epithelial origin, including the cardiomyocytes [90]. HGF is also shown to promote cardiomyocyte differentiation, proliferation, and regeneration [91], and to protect from myocardial infarction [92] and/or ischemia/reperfusion injury [93]. Post-infarction treatment with HGF improves left ventricular remodeling and heart function [94]. In addition, HGF also improves heart functionality and promotes the proliferation of myocardial progenitor cells in doxorubicin-induced cardiomyopathy [31].

Until now, only a few studies have reported on miR-499 in cardiomyocytes. The miR-499 is reported to be expressed specifically in the heart and skeletal muscles of humans and mice [95,96,97], contributing to the cardiac differentiation of mesenchymal stem cells [98], late-stage cardiomyocyte differentiation [97], and the expression of the voltage-dependent calcium channel β-2 subunit [99]. A number of studies have indicated that miRNAs play a role in the regulation of many biological processes in the cardiomyocytes (migration, cell proliferation, apoptosis, differentiation, etc.).

Reg IV and Hgf were revealed in this study to function as anti-apoptotic/growth-promoting factors in cardiomyocytes, and both Reg IV and Hgf were up-regulated in cardiomyocytes in the IH condition, but not in the SH. This suggests that both Reg IV and Hgf protect cardiomyocytes from cell death/stress due to decreased oxygen concentrations in IH, but not in SH. The possible protection of cardiomyocytes from decreased oxygen concentrations may be achieved by the expression of Reg IV/Hgf or by the inhibition of miR-499.

In conclusion, this study revealed that the gene expressions of *Reg IV* and *Hgf* were increased via the downregulation of the miR-499 level in IH-treated cardiomyocytes and that both Reg IV and Hgf acted as anti-apoptotic factors in the cardiomyocytes. It is suggested that, in SAS patients, the upregulation of *REG IV* and *HGF* may function against the apoptosis of cardiomyocytes, leading to the maintenance of cardiac functions, and that miR-499 could play a crucial role in the regulation of these gene expressions.

## 4. Materials and Methods

### 4.1. Cell Culture

Rat H9c2 cardiomyocytes were purchased from the American Type Culture Collection (Manassas, VA, USA). The cells were maintained in Dulbecco’s Modified Eagle Medium (DMEM) (FUJIFILM Wako Pure Chemical Corporation, Osaka, Japan) containing 10% (*v*/*v*) fetal calf serum (FCS), 100 units/mL penicillin G (FUJIFILM Wako), and 100 µg/mL streptomycin (FUJIFILM Wako). Mouse embryonic carcinoma P19.CL6 cells were purchased from RIKEN BioResource Research Center (Tsukuba, Japan). The cells were grown in Minimum Essential Medium Alpha Modification (MEMα) (FUJIFILM Wako) medium containing 10% (*v*/*v*) FCS, 100 units/mL penicillin G, and 100 µg/mL streptomycin. For the differentiation experiments, 3.7 × 10^5^ cells/0.5 mL were seeded in a 24-well cell culture plate with MEMα medium containing 1% DMSO for 10 days to induce cardio-myogenesis as described [100]. The cells were kept at 37 °C, 5% CO_2_, and 95% humidity, and the medium was changed every day. Cells were exposed to either normoxia (21% O_2_, 5% CO_2_, and balanced N_2_), intermittent hypoxia (IH: 70 cycles of 5 min sustained hypoxia [1% O_2_, 5% CO_2_, and balanced N_2_] and 10 min normoxia), or sustained hypoxia (1% O_2_, 5% CO_2_, and balanced N_2_) using a custom-designed, computer-controlled incubation chamber attached to an external O_2_-CO_2_-N_2_ computer-driven controller (O_2_ programmable control, 9200EX, Wakenbtech CO., Ltd., Kyoto, Japan), as described [3,101,102,103,104,105,106,107]. These conditions are similar to the conditions reported in patients with severe degrees of SAS: in severe cases of SAS, patients are repeatedly exposed to severe hypoxemia followed by mild hypoxemia or normoxia (i.e., IH). We previously reported that the magnitude of IH expressed by SpO_2_ fluctuated between 75–98% and 50–80% in SAS [3,4], which was almost equivalent to the medium condition in the present study.

### 4.2. Real-Time RT-PCR

Total RNA was isolated using an RNeasy plus mini kit (Qiagen, Hilden, Germany) from H9c2 and P19.CL6 cells, and cDNA was synthesized from total RNA as a template using a High Capacity cDNA Reverse Transcription kit (Applied Biosystems, Foster City, CA, USA), as described [73,74,75,79,82,101,102,103,104,105,106,107,108,109,110]. Real-time PCR was performed using SYBR^®^ Fast qPCR kit (KAPA Biosystems, Boston, MA, USA) and a Thermal Cycler Dice Real Time System (Takara Bio, Kusatsu, Japan). All the PCR primers were synthesized by Nihon Gene Research Laboratories, Inc. (NGRL; Sendai, Japan), and the primer sequences for each primer set are described in Table 1. PCR was performed with an initial step of 3 min at 95 °C, followed by 40 cycles of 3 s at 95 °C and 20 s at 60 °C for *rat insulinoma gene (Rig)/ribosomal protein S15* (*RpS15*), *Il-6, Il-8*, *Il-17A*, *Il-18*, *Il-33*, *Tgfβ1*, *Ccl2, Cxcl12, Tnfα*, *Vegf-A*, *Flt-1*, *Flk-1*, *Cd38*, *Reg I*, *Reg II*, *PAP II/Reg IIIα*, *PAP I/Reg IIIβ*, *PAP III/Reg IIIγ*, *Reg IIIδ*, *Reg IV*, *Extl3* (*Reg receptor*), *Hgf*, and *c-Met* (*Hgf receptor*). The mRNA expression levels were normalized to the mRNA level of *Rig/RpS15*, as described [75,79,85,104,105,106,107]. For miR, total RNA, including miRNA, was isolated from P19.CL6 cells using the miRNeasy mini kit (Qiagen) according to the manufacturer’s instructions. An equal amount of DNase-treated RNA was Poly-A-tailed using a Mir-X^TM^ miRNA first-strand synthesis kit (Clontech Laboratories, Inc., Mountain View, CA, USA) according to the manufacturer’s protocol. The conditions for PCR were 95 °C for 10 s, followed by 45 cycles of amplification (95 °C, 5 s, 60 °C, 20 s). U6 small nuclear RNA was used as an endogenous control for miRNA, as previously described [75,79,105,106].

### 4.3. Measurement of Mouse Reg IV and Hgf in Culture Medium by ELISA

Differentiated P19.CL6 cardiomyocytes were exposed to either normoxia or IH for 24 h. The culture medium was collected, and the concentrations of mouse Reg IV and Hgf were measured using the ELISA Kit for mouse Reg IV (Cloud-Clone Corp., Katy, TX, USA) [75] and for mouse Hgf (R&D Systems, Inc., Minneapolis, MN, USA), respectively.

### 4.4. Measurement of Viable Cell Numbers by Tetrazolium Salt Cleavage

P19.CL6 cells differentiated to cardiomyocytes (7.4 × 10^4^ cells/100 µL in 96-well plate) were incubated at 37 °C overnight, and the medium was replaced with fresh MEMα + 10% FCS just before the addition of recombinant mouse Reg IV protein (R&D Systems) or mouse Hgf (R&D Systems). After a 24-h treatment with Reg IV or Hgf, the viable cell numbers were determined by a Cell Counting kit-8 (Dojindo Laboratories, Mashiki-machi, Japan), according to the manufacturer’s instructions. Briefly, WST-8 solution was added to cells in 96-well plates, and the cells were incubated at 37 °C for 30 min. The optical density of each well was read at 450 nm (reference wave length at 650 nm) using a Sunrise^TM^ microplate reader (Tecan, Männedorf, Switzerland), as described [36,58,75,79,85,108].

### 4.5. Measurement of Apoptosis

P19.CL6 cells (2.5 × 10^4^ cells/100 µL in 96-well plate) were incubated and differentiated into cardiomyocytes by incubating with 1% DMSO for 10 days [100]. After the cells were differentiated into cardiomyocytes, they were exposed to normoxia, IH, or SH with/without 0.1 ng/mL recombinant Reg IV (R&D Systems) and 0.1 ng/mL recombinant mouse Hgf (R&D Systems) for 24 h, and apoptosis was detected by the TUNEL method using an apoptosis screening kit (FUJIFILM Wako). The optical density of each well was read at 490 nm (reference wave length at 650 nm) using a Sunrise^TM^ microplate reader (Tecan), as described [36,58,108,110].

### 4.6. Measurement of Replicative DNA Synthesis

IdU solution was added to the culture medium of differentiated P19.CL6 cells (2.0 × 10^4^ cells/100 µL in 96-well plate). After 1 h incubation in the presence of recombinant mouse Reg IV (0.1 ng/mL) and/or recombinant mouse Hgf (0.1 ng/mL), IdU incorporation was measured using a DNA-IdU Labeling and Detection kit (Takara Bio) as described [53,108,110]. The optical density of each well was read at 490 nm (reference wave length at 650 nm) using a Sunrise^TM^ microplate reader (Tecan).

### 4.7. MiR-499 Mimic Transfection

MiR-499 mimic (5′-UUAAGACUUGCAGUGAUGUuu-3′, 5′-ACAUCACUGCAAGUCUUAAuu-3′; 14–32 of NR_030757.1) and non-specific control RNA (miR-499 mimic NC) (5′-UUCUCCGAACGUGUCACGUtt-3′, 5′-ACGUGACACGUUCGGAGAAtt-3′) were synthesized by NGRL and introduced into differentiated P19.CL6 cardiomyocyte using Lipofectamine^®^ RNAiMAX Transfection Reagent (Invitrogen, Waltham, MA, USA) [75,79,104,105,106] just before IH/normoxia exposure. The mRNA levels of *Reg IV* and *Hgf* were measured by real-time RT-PCR, as described [58,75,79,101,104,105,106].

### 4.8. Construction of Reporter Plasmid and Luciferase Assay

Reporter plasmids were prepared by inserting the promoter fragments of mouse *Reg IV* (−2008–+29) and rat *Hgf* (−1336–+59) upstream of a firefly luciferase reporter gene in the pGL4.17[*luc2*/Neo] vector (Promega, Madison, WI) and pGL3-Basic (Promega) [36], respectively. The reporter plasmids were transfected into mouse P19.CL6 cells differentiated into cardiomyocytes using Lipofectamine^®^ 3000 (Invitrogen), as described [36,73,74,75,79,82,102,105,106,107,108,109]. The cells were exposed to either 70 cycles/24 h of IH, mimicking the cardiomyocytes of SAS patients, or normoxia for 24 h. After the cells were exposed to IH, they were lysed, and promoter activities were measured. The cells were harvested, and cell extracts were prepared in an Extraction Buffer (0.1 M potassium phosphate, pH 7.8/0.2% Triton X-100; Life Technologies, Carlsbad, CA, USA). To monitor transfection efficiency, pCMV•SPORT-βgal plasmid (Life Technologies) was co-transfected in all experiments at a 1:10 dilution. Luciferase activity was measured using a PicaGene luciferase assay system (Toyo-ink, Tokyo, Japan) and was normalized by the β-galactosidase activity as described previously [36,58,73,74,75,79,82,102,103,104,105,106].

### 4.9. Data Analysis

Results are expressed as mean ± SE. Statistical significance was determined by Student’s *t*-test using GraphPad Prism software (GraphPad Software, La Jolla, CA, USA).

## Figures and Tables

**Table 1:** PCR primers for real-time RT-PCR.

Target mRNA	Primer Sequence (Position)
Rat	
*Il-6*	5′-AAGTCGGAGGCTTAATTACATATGTTC-3′ (NM_012589.2: 213–239)
	5′-TGCCATTGCACAACTCTTTTCT-3′ (NM_012589.2: 260–281)
*Il-17A*	5’-TCTCCAGAACGTGAAGGTC-3’ (NM_001106897.1: 187–205)
	5’-AAGTGGAACGGTTGAGGTAG-3’ (NM_001106897.1: 262–281)
*Il-18*	5′-ATATCGACCGAACAGCCAAC-3′ (AJ222813.1: 212–231)
	5′-TAGGGTCACAGCCAGTCCTC-3′ (AJ222813.1: 281–300)
*Il-33*	5′-CAAAGATATCTGCCATGTCTAC-3′ (NM_001014166.1: 177–198)
	5′-AAGCAGGGATCTCTTCCTAG-3′ (NM_001014166.1: 329–348)
*Tnfα*	5′-CCCAGACCCTCACACTCAGATCAT-3′ (NM_012675.3: 368–391)
	5′-GCAGCCTTGTCCCTTGAAGAGAA-3′ (NM_012675.3: 566–588)
*Tgfβ1*	5′-GCTAATGGTGGACCGCAACAAC-3′ (NM_021578.2: 478–499)
	5′-CAGCAGCCGGTTACCAAG-3′ (NM_021578.2: 689–706)
*Cxcl12*	5′-GCATCAGTGACGGTAAGC-3′ (AF217564.1: 103–120)
	5′-GAAGGGCACAGTTTGGAG-3′ (AF217564.1: 208–225)
*Ccl2*	5′-CCCAATGAGTCGGCTGGAG-3′ (NM_031530.1: 204–222)
	5′-TAAGGCATCACATTCCAAAT-3′ (NM_031530.1: 527–546)
*Vegf-A*	5′-TTGAGACCCTGGTGGACATC-3′ (NM_031836.3: 1175–1194)
	5′-GGATCTTGGACAAACAAATGC-3′ (NM_031836.3: 1536–1556)
*Flt-1*	5′-TCCCTCAGCCTACCATCAAG-3′ (NM_019306.2: 1611–1630)
	5′-GAGAGTCAGCCACCACCAAT-3′ (NM_019306.2: 1798–1817)
*Flk-1*	5′-ACAGCATCACCAGCAGTCAG-3′ (NM_013062.2: 3132–3151)
	5′-CCAAGAACTCCATGCCCTTA-3′ (NM_013062.2: 3280–3299)
*Cd38*	5′-GAAAGGGAAGCCTACCACGAA-3′ (NM_013127.1: 166–186)
	5′-GCCGGAGGATTTGAGTATAGATCA-3′ (NM_013127.1: 219–242)
*Reg I*	5′-GGACACTGGGTATCCTAACAATTCC-3′ (M18962.1: 424–448)
	5′-CTCTCCATTTCTTGTATCCTGAGTTTG-3′ (M18962.1: 477–503)
*PAP I*	5′-AAAATACCCTCTGCACGCATTAG-3′ (NM_053289.1: 153–175)
	5′-GGGCATAGCAGTAGGAGCCATA-3′ (NM_053289.1: 198–219)
*Reg III/PAP II*	5′-CCAGAAGGCAGTGCCCTCTA-3′ (L10229.1: 240–259)
	5′-GCAGTAAGAACGATAAGCCTTGGA-3′ (L10229.1: 283–306)
*PAP III*	5′-TGTGCCCACTTCACGTATCAG-3′ (NM_173097.1: 121–141)
	5′-GGCATAGCAATAGGAGCCATAGG-3′ (NM_173097.1: 162–184)
*Reg IV*	5′-CTGCTGAGCTGGGTAGCTGGCCC-3′ (NM_001004096.1: 31–53)
	5′-TTTATCCTTGGGGTTCATCTCAG-3′ (NM_001004096.1: 386–408)
*Extl3*	5′-CAATCGGTTCTTGCCCTGG-3′ (NM_020097.2: 2182–2200)
	5′-GGAAGTTCATGGCGATATCC-3′ (NM_020097.2: 2500–2519)
*Hgf*	5′-GGCTGAAAAGATTGGATCAGGAC-3′ (NM_017017.2: 2131–2153)
	5′-ATCCACGACCAGGAACAATG-3′ (NM_017017.2: 2221–2240)
*c-Met*	5′-CAGACGCCTTGTATGAAGT-3′ (NM_031517.2: 3929–3947)
	5′-CATAAGTAGCGTTCACATGG-3′ (NM_031517.2: 4053–4072)
*Rig/RpS15*	5′-ACGGCAAGACCTTCAACCAG-3′ (NM_017151.2: 314–333)
	5′-ATGGAGAACTCGCCCAGGTAG-3′ (NM_017151.2: 363–383)
Mouse	
*Il-6*	5′-TTCCATCCAGTTGCCTTCTTG-3′ (NM_031168.2: 103–123)
	5′-GAAGGCCGTGGTTGTCACC-3′ (NM_031168.2: 135–153)
*Il-8*	5′-CAGAAAGGAAGTGATAGCAGTCCCA-3′ (NM_011339.2: 211–235)
	5′-CAAAGTGTCTAGAGGTCTCCCGAA-3′ (NM_011339.2: 441–464)
*Il-17A*	5’-TTTAACTCCCTTGGCGCAAAA-3’ (NM_010552.3: 217–237)
	5’-CTTTCCCTCCGCATTGACAC-3’ (NM_010552.3: 362–381)
*Il-18*	5′-ACTGTACAACCGCAGTAATACGG-3′ (NM_008360.2: 714–736)
	5′-TCCATCTTGTTGTGTCCTGG-3′ (NM_008360.2: 1013–1032)
*Tnfα*	5′-CGTCAGCCGATTTGCTATCT-3′ (NM_013693.3: 638–657)
	5′-CGGACTCCGCAAAGTCTAAG-3′ (NM_013693.3: 824–843)
*TGFβ*	5′-CCACCTGCAAGACCATCGAC-3′ (NM_011577.2: 959–978)
	5′-CTGGCGAGCCTTAGTTTGGAC-3′ (NM_011577.2: 1029–1049)
*Cxcl12*	5′-GCGCTCTGCATCAGTGAC-3′ (NM_021704.3: 164–181)
	5′-TTTCAGATGCTTGACGTTGG-3′ (NM_021704.3: 246–265)
*Ccl2*	5′-TTCACCAGCAAGATGATCCCA-3′ (NM_011333.3: 197–217)
	5′-TCCTTCTTGGGGTCAGCACA-3′ (NM_011333.3: 308–327)
*Vegf-A*	5′-AGTGGCTTACCCTTCCTCATCTT-3′ (NM_001025250.3: 2707–2729)
	5′-CGGGTCCTGCCCCATT-3′ (NM_001025250.3: 2750–2765)
*Flt-1*	5′-GAGGAGGATGAGGGTGTCTATAGGT-3′ (NM_010228.4: 2447–2471)
	5′-GTGATCAGCTCCAGGTTTGACTT-3′ (NM_010228.4: 2540–2562)
*Flk-1*	5′-GCATCACCAGCAGCCAGAG-3′ (NM_010612.3: 3175–3193)
	5′-GGGCCATCCACTTCAAAGG-3′ (NM_010612.3: 3483–3501)
*Cd38*	5′-ACAGACCTGGCTGCCGCCTCTCTAG-3′ (NM_007646.5: 102–126)
	5′-GGGGCGTAGTCTTCTCTTGTGATGT-3′ (NM_007646.5: 378–402)
*Reg I*	5′-AAGGAGAGTGGCACTACAGACG-3′ (NM_009042.2: 333–354)
	5′-GTATTGGGCATCACAGTTGTCA-3′ (NM_009042.2: 521–542)
*Reg II*	5′-ACAGCCAAGGCCAGGTAGCT-3′ (NM_009043.2: 127–146)
	5′-GGGCAGTTGATTTTGGCAGA-3′ (NM_009043.2: 183–202)
*Reg IIIα*	5′-GGATTGGGCTCCATGATCC-3′ (NM_011259.1: 386–404)
	5′-TCAGCACATCGGAGTTACTCCA-3′ (NM_011259.1: 442–463)
*Reg IIIβ*	5′-TGCCTTGTTTCAGATACCACAGA-3′ (NM_011036.1: 187–209)
	5′-GGTGTCCTCCAGGCCTCTTT-3′ (NM_011036.1: 238–257)
*Reg IIIγ*	5′-GGTAACAGTGGCCAATATGTATGG-3′ (NM_011260.2: 318–341)
	5′-CCACCTCTGTTGGGTTCATAG-3′ (NM_011260.2: 368–388)
*Reg IIIδ*	5′-GTGTTGCCTGATGTCCCTTTC-3′ (NM_013893.2: 102–122)
	5′-CAGCTGATGCGTGGAGAAGAC-3′ (NM_013893.2: 156–176)
*Reg IV*	5′-CGTGCGGCTACTCTTACTGCT-3′ (NM_026328.2: 179–199)
	5′-AGCTGGGTCTCAAGATATCGCT-3′ (NM_026328.2: 228–249)
*Extl3*	5′-CAATCGGTTCTTGCCCTGG-3′ (NM_018788.3: 2842–2860)
	5′-GGAAGTTCATGGCGATATCC-3′ (NM_018788.3: 3160–3179)
*Hgf*	5′-GGCTGAAAAGATTGGATCAGGAC-3′ (NM_010427.5: 2166–2188)
	5′-ATCCACGACCAGGAACAATG-3′ (NM_010427.5: 2256–2275)
*c-Met*	5′-TCGGACAGAGTTTACCACG-3′ (NM_008591.2: 1600–1618)
	5′-TCCAGGAGGAAGTTCACAT-3′ (NM_008591.2: 1779–1797)
*Dicer*	5′-ATGCAAAAAGGACCGTGTTC-3′ (NM_148948.2: 524–543)
	5′-CAAGGCGACATAGCAAGTCA-3′ (NM_148948.2: 698–717)
*Drosh*	5′-CTCTTTCCCACCCAGTGCTA-3′ (NM_001130149.1: 844–865)
	5′-TGGTCGTCGTAGTGCTTGAG-3′ (NM_001130149.1: 947–966)
*Rig/RpS15*	5′-ACGGCAAGACCTTCAACCAG-3′ (NM_009091.2: 343–362)
	5′-ATGGAGAACTCGCCCAGGTAG-3′ (NM_009091.2: 392–412)
*miR-499*	5′-TTAAGACTTGCAGTGATGTTT-3′ (NR_030757.1: 10–28)
	5′-GAACATGTCTGCGTATCTC-3′ (NR_030757.1: 36–53)
*U6*	5’-CGCTTCGGCAGCACATATAC-3′ (XR_004940589.1: 6–25)
	5′-AAATATGGAACGCTTCACGA-3′ (XR_004940589.1: 86–105)

## Data Availability

The data are available on request from the authors.

## Abbreviations

Ccl2	C-C motif chemokine 2
Cd38	Cluster of differentiation 38
c-Met	Tyrosine-protein kinase Met
Cxcl12	C-X-C motif chemokine 12
CVD	Cardiovascular disease
DICER	Endoribonuclease Dicer
DMEM	Dulbecco’s Modified Eagle Medium
DROSHA	Ribonuclease type III
ELISA	Enzyme-linked Immunosorbent assay
Extl3	Exostosin-like 3
FCS	Fetal calf serum
Flk-1	Fetal liver kinase receptor 1
Flt-1	Fms-like tyrosine kinase 1
HGFIdU	Hepatocyte growth factor5-Iodo-2ʹ-deoxyuridine
IH	Intermittent hypoxia
Il	Interleukin
MEMα	Minimum Essential Medium Alpha Modification
miR	MicroRNA
PAP	Pancreatitis associated protein
Reg	Regenerating gene
RpS15	Ribosomal protein S15
RT-PCR	Reverse transcriptase-polymerase chain reaction
SAS	Sleep apnea syndrome
SH	Sustained hypoxia
TGFβ1	Transforming growth factor β1
Tnfα	Tumor necrosis factor-α
TUNEL	TdT-mediated dUTP nick end labeling
Vegf-A	Vascular endothelial growth factor A
WST-8	2-(2-methoxy-4-nitrophenyl)-3-(4-nitrophenyl)-5-(2,4-disulfophenyl)-*2H*-tetrazolium monosodium salt
